# Correlation between NK function and response to trastuzumab in metastatic breast cancer patients

**DOI:** 10.1186/1479-5876-6-25

**Published:** 2008-05-16

**Authors:** Alessandra Beano, Elena Signorino, Andrea Evangelista, Davide Brusa, Marinella Mistrangelo, Maria Antonia Polimeni, Rosella Spadi, Michela Donadio, Libero Ciuffreda, Lina Matera

**Affiliations:** 1 Dept of Medical Oncology, Molinette's Hospital, Turin, Italy; 2 Laboratory of Tumour Immunology, Dept of Internal Medicine, University of Turin, Italy; 3 Unit of Cancer Epidemiology, S. Giovanni Battista, Hospital and CPO Piemonte, Turin, Italy

## Abstract

**Background:**

Trastuzumab is a monoclonal antibody selectively directed against Her2 and approved for the treatment of Her2 overexpressing breast cancer patients. Its proposed mechanisms of action include mediation of antibody-dependent cellular cytotoxicity (ADCC) by triggering FcγRIII on natural killer (NK) cells. This study addresses the correlation between overall NK function and trastuzumab's clinical activity.

**Subjects and methods:**

Clinical and immunological responses were assessed in 26 patients receiving trastuzumab monotherapy as maintenance management after chemotherapy (8 mg/kg load and then standard doses of 6 mg/kg every 3 weeks). Cytotoxic activity against the MHC class I-negative standard NK target K562 cell line and HER2-specific ADCC against a trastuzumab-coated Her2-positive SKBR3 cell line were assessed in peripheral blood mononuclear cells (PBMC) harvested after the first standard dose. After six months, seventeen patients were scored as responders and nine as non-responders according to the RECIST criteria, while Progression-Free Survival (PFS) was calculated during a 12 months follow-up.

**Results:**

The responders had significantly higher levels of both NK and ADCC activities (p < 0.05) that were not different from those of eleven normal controls. The NK activity of the non-responders was significantly (p < 0.05) lower than that of the normal controls. At twelve months, there was a marked correlation between PFS and NK activity only. PFS was significantly longer in patients with high levels of NK activity, whereas its pattern was unrelated to high or low ADCC activity.

**Conclusion:**

One of the mechanisms of action of trastuzumab is NK cell-mediated ADCC lysis of the Her2-positve target cell. We show here that its potency is correlated with the short-term response to treatment, whereas longer protection against tumor expansion seems to be mediated by pure NK activity.

## Background

Breast cancer is the second most common cancer in the world (after lung cancer), and a major cause of cancer-related death in women [1].

Human Epidermal Growth Factor Receptor 2 (Her2), a member of the ErbB family that plays an important role in promoting oncogenic transformation and tumour growth [2] is overexpressed in 20–30% of patients [3,4]. This overexpression correlates with poor prognosis, including high risk of recurrence, metastases and reduced overall survival [2,5,6].

Trastuzumab is a humanised monoclonal antibody that selectively targets Her2. Its use is approved for the treatment of women with Her2-overexpressing breast cancer, as determined by an accurate and validated assay [7-9]. Enhancement of Her2 degradation, inhibition of cell cycle progression via inhibition of the mitogen-activated protein kinase pathway and suppression of antiapoptotic phosphatylinositol 3-kinase and Akt pathways have been shown to follow its binding to Her2-overexpressing cells [10-15]. However, only 25–30% [10] or 18% [15] of patients with Her2 over-expression respond to treatment. There is thus a need to find predictive response markers in order to select patients likely to benefit from this treatment and spare others its adverse effects [15]. Reliable markers may emerge from evaluation of all the players involved in trastuzumab-mediated tumor regression.

Both cytostatic and cytolytic mechanisms account for the clinical effect of trastuzumab. Her2-related cytotoxicity includes complement-mediated and antibody-dependent cellular cytotoxicity (ADCC) mediated by FcγRIII [16-22]. Cell subsets that mediate ADCC include neutrophils, monocytes, and natural killer (NK) cells. NK cells are cytotoxic in two ways. First, they spontaneously lyse virus- infected or transformed cells in the absence of prior sensitization. Since this activity is under the dominant control of inhibitory receptors (iNKRs) that bind class I human leukocyte antigen (HLA), it is thought to be only effective against tumor cells that lack MHC class I or present a dominant-activating ligand. Second, NK cells recognize and kill antibody-coated target cells during ADCC [19-23]. Increased number of NK cells at the tumor site after trastuzumab and a correlation between NK tumor infiltration and clinical response [22] are convincing evidence of their participation in tumor clearance. The present study addresses the significance of NK cells in the mechanism of action of trastuzumab by comparing their functional state and the clinical outcome in metastatic breast cancer patients.

## Methods

### Patients

Twenty-six metastatic breast cancer patients (Table 1) received trastuzumab as a single agent after chemotherapy in the form of an 8 mg/kg load followed by 6 mg/kg standard doses every 3 weeks for 1 year, or until evidence of disease progression or unacceptable toxicity. NK and ADCC activities were assessed in occasion of the first standard dose. Clinical response was evaluated radiologically and classified according to the RECIST criteria. Patients experiencing CR, PR and SD were considered responders, as opposed to PD patients who were considered non-responders (Table 1).

**Table 1:** Characteristics of patients

*Patient #*	*Type of mts*	*No. of mts*	*Response*
999	Visceral^1^	> 3	DP
1025	Visceral	< 3	CR
1080	Visceral	> 3	DP
1081	Visceral	< 3	DP
1083	Visceral	> 3	PR
1095	Visceral	< 3	CR
1125	Visceral	> 3	DP
1126	Visceral	< 3	SD
1102	Non-visceral^2^	> 3	CR
1103	Non-visceral	< 3	SD
1110	Non-visceral	< 3	CR
1121	Non-visceral	> 3	DP
1123	Non-visceral	< 3	SD
1124	Non-visceral	< 3	CR
1019	Mixed^3^	> 3	PR
1020	Mixed	> 3	SD
1021	Mixed	> 3	PR
1026	Mixed	> 3	DP
1082	Mixed	> 3	DP
1104	Mixed	> 3	PR
1105	Mixed	> 3	SD
1116	Mixed	> 3	DP
1270	Visceral	< 3	RC
1276	Mixed	> 3	PR
1279	Non-visceral	< 3	PR
1280	Visceral	> 3	PD

^1^Visceral mts: liver, lungs, CNS

^2^Non-visceral metastases (mts): bones, locoregional lymph nodes, skin

^3^Mixed: visceral and non-visceral

DP: disease progression; CR: complete remission; PR: partial remission; SD: stable disease.

### Cytokines and Antibodies

Recombinant human IL-2 was purchased from Chiron (Milan, Italy). The humanized anti-Her2 MoAb trastuzumab was kindly provided by Genentech Inc. (San Francisco, California, USA).

### Cell lines

The human breast adenocarcinoma lines SKBR3 and MCF7 were grown as adherent cells and used as Her2-positive and Her2-negative lines, respectively. The non-adherent leukaemia cell line K562 [24] was used as an MHC class I-negative, NK susceptible target. Its NK susceptibility was tested in pilot and concurrent experiments against the standard NK-resistant DAUDI cell line. Culture medium was RPMI 1640 (Life Technologies Ltd, Paisley, Scotland, UK) supplemented with 20% (SKBR3 and MCF7) or 10% (K562) heat inactivated fetal calf serum (FCS), 1% L-glutamine, 1% penicillin and streptomycin (Sigma-Aldrich, Milan, Italy).

### Isolation of PBMC

Peripheral blood mononuclear cells (PBMC) obtained after Ficoll-Hypaque density centrifugation of venous blood of patients and age-matched normal donors were washed in RPMI 1640 supplemented with 5 mM EDTA (Sigma-Aldrich) and 2% heat inactivated FCS and resuspended in RPMI 10% FCS.

### ADCC and NK cytotoxicities

To evaluate ADCC, the SKBR3 and MCF7 breast cancer cell lines were incubated with 10 μg/ml trastuzumab at 4°C for 30 minutes. The mAb excess was removed by washing at 4°C to prevent capping and Ag-Ab complexes endocytosis. The cells were used as targets in a ^51^Cr release cytotoxicity assay, where the mAb uncoated K562 line was used to evaluate NK activity. This assay was conducted as previously described [25]. Briefly, targets cells were incubated with 100 μCi Na_2 _^51^CrO_4_(Perkin Elmer, Milan, Italy), for 1 h, washed and added (5 × 10^3^/well of a 96-well V-bottomed plate) to different numbers of effector cells to obtain decreasing effector to target (E:T) ratios (50:1, 25:1 and 12:1), or cultured with medium alone or TritonX-100. Plates were incubated at 37°C for 4 h, and the supernatant (100 μl) was harvested for quantification in a γ-counter. Triplicate wells were set up for each E:T dilution and the percentage of lysis was calculated according to the formula: (experimental release – spontaneous release)/(maximal release – spontaneous release) × 100, where experimental release represents the mean counts per minute (cpm) for the target cells in the presence of effector cells, spontaneous release represents the mean cpm for target cells incubated without effector cells, and maximal release represents the mean cpm for target cells incubated with TritonX-100. Experiments with spontaneous releases higher than 10% (for the non-adherent cell line K562) or 20% (for the adherent cell lines SKBR3 and MCF7) were not considered.

### Statistics

Responder and non-responder groups were compared using unpaired Student's t-test and Welch's t-test for unequal variance. Receiver operating characteristic (ROC) curve analysis was performed to detect optimal cut-off points of NK and ADCC to evaluate the clinical response at six months. For Progression-Free Survival (PFS) analysis, Kaplan-Meier curves were produced and compared with Log-Rank tests. The statistical package STATA 9.2 was used for all statistical tests and a two-tailed p-value of less than .05 was considered significant.

## Results

### Expression of Her2 on SKBR3 and MCF7 cell lines

**Figure 1:**
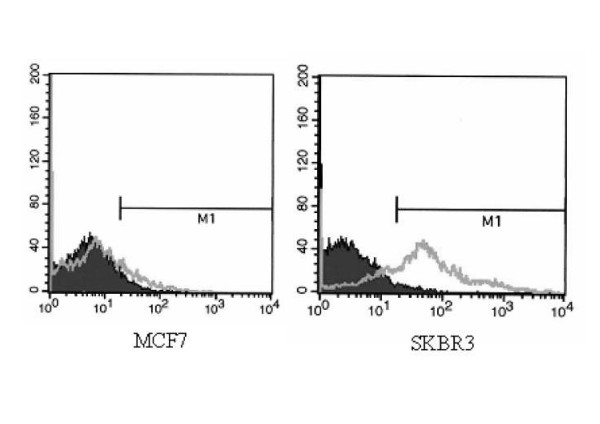
**Different Her2 expression of the ADCC target cell lines**. The human breast carcinoma cell lines MCF7 and SKBR3 were stained with the humanized anti-Her2 MoAb trastuzumab (Genentech Inc. San Francisco, California, USA) and analysed by flow cytometry.

Since conflicting assessment have been made of the expression of Her2 on MCF7 cells [17,19,22], we have unequivocally stated the Her2 expression of our two lines. Results show that the SKBR3 is highly Her2-positive, whereas the MCF7 line is substantially Her2-negative (Fig. 1).

### In vitro cytotoxicity of NK targets and breast tumor targets by patient's PBMC correlates with their clinical response at six months

**Figure 2:**
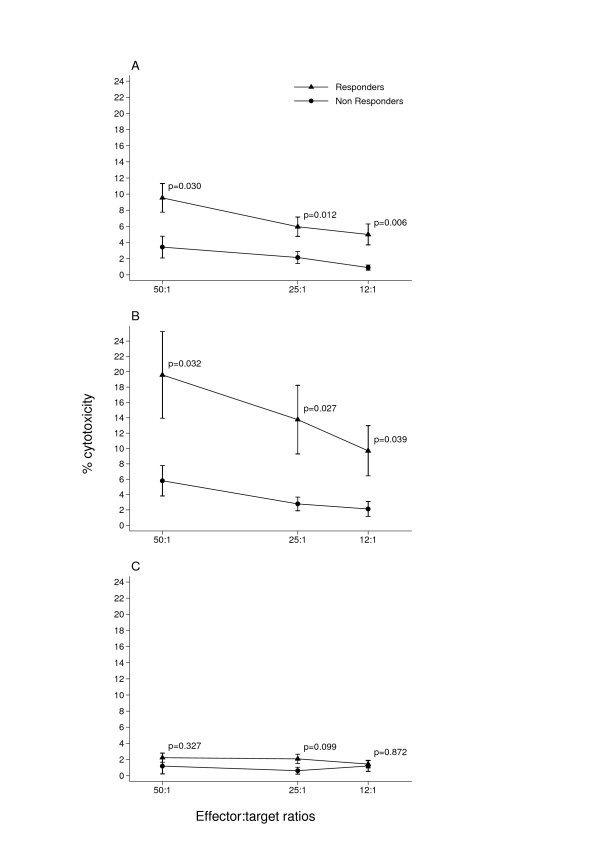
**NK and ADCC profile of patients classified as responders and non-responders after a six-month trastuzumab regimen**. NK and ADCC activities of PBMC were tested at the start of trastuzumab therapy against the target K562 cell line (A) and the trastuzumab-coated Her2-positive cell line SKBR3 (B) in a ^51^Cr release assay. The trastuzumab-coated Her2-negative cell line MCF7 (C) was used as control for ADCC. Values of NK (A) and ADCC (B) cytotoxicity were always significantly higher in responders at all three E:T ratios.

**Figure 3:**
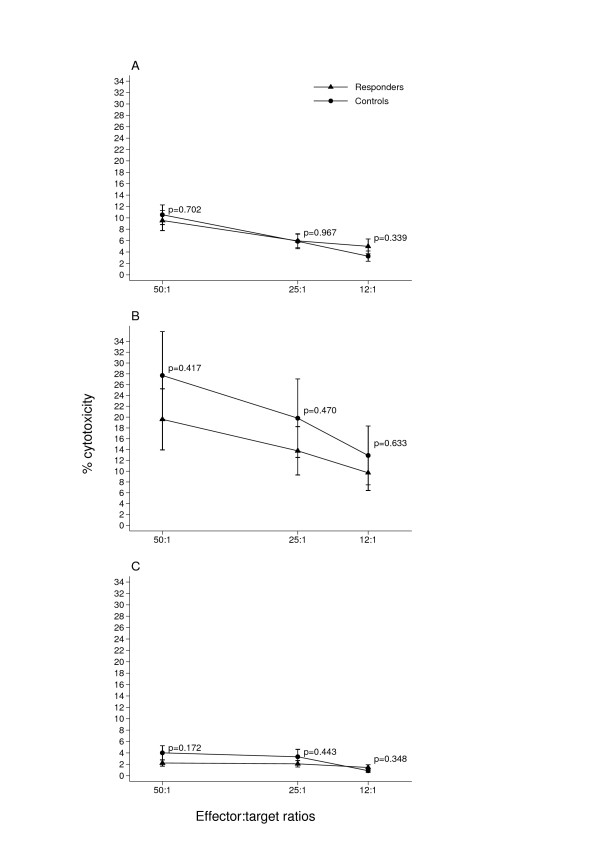
**Responders have normal  NK and ADCC activities**. NK and ADCC activities were assessed as described in legend to Fig. 2. Values (A and B) were not significantly different in responders compared to 11 normal donors at all three E:T ratios. As expected, negligible ADCC activity was found both in patients and normal donors against the Her2-negative cell line MCF7 (C).

**Figure 4:**
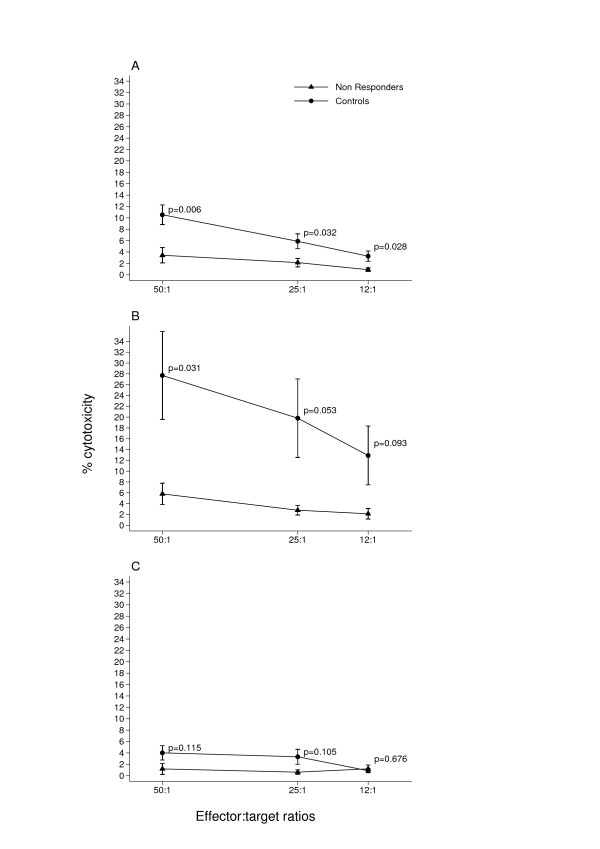
**Non-responder patients have defective NK activity**. NK and ADCC activities were assessed as described in legend to Fig. 2. Values of NK activity (A) were significantly lower in patients compared to normal donors at three E:T ratios. ADCC activity (B) was significantly lower in patients only at the highest E:T ratio. As expected, negligible ADCC activity was found both in patients and normal donors against the Her2-negative cell line MCF7 (C).

Patients undergoing trastuzumab monotherapy after trastuzumab-chemotherapy association were tested for NK activity against the NK target K562 and for ADCC activity against the trastuzumab-coated Her2-positive cell line SKBR3 in a ^51^Cr release assay. The Her2-negative cell line MCF7 was employed as a negative control for the ADCC. After six months seventeen patients were classified as responders (CR, PR and SD) and nine as non-responders (PD) according to the RECIST criteria (Table 1). Both NK (Fig. 2A). and ADCC (Fig. 2B). activities were two-fold higher in the responders. This difference was significant at all three E:T ratios. As expected, ADCC against the Her2-negative cell line MCF7 was much lower than that against the SKBR3 cell line (Fig. 2C). Furthermore, the NK and ADCC activities of responders were not different from those of eleven normal controls (Fig 3A and 3B), whereas the NK activity of the non-responders was significantly lower (Fig. 4A) and their ADCC activity (Fig. 4B) was clearly lower than normal, though only significant at the highest E:T ratio.

### Correlation between NK and ADCC activities and PFS

**Figure 5:**
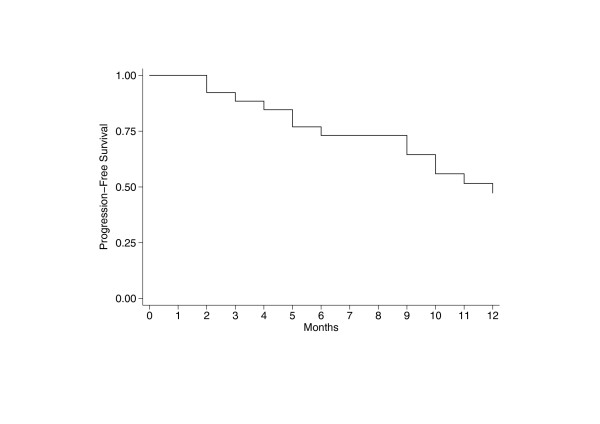
**Global time to tumor progression at twelve months**. The Kaplan-Meier PFS curve shows that the percentage of progression-free patients is 75% at six months from start of Trastuzumab therapy and declines to 50% at twelve months.

**Figure 6:**
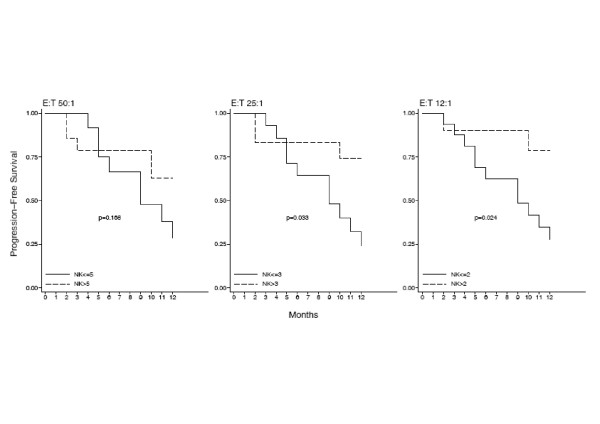
**Time to tumor progression is correlated with the NK activity**. A cut-off of 5%. 3% and 2% cytotoxicity was chosen to discriminate between high and low NK levels at the three E:T ratios of 50:1, 25:1 and 12:1 respectively and a stratified PFS curve was created from these values. The figure shows that higher NK values are significantly (Log-Rank test p values) correlated with longer time to tumor progression.

**Figure 7:**
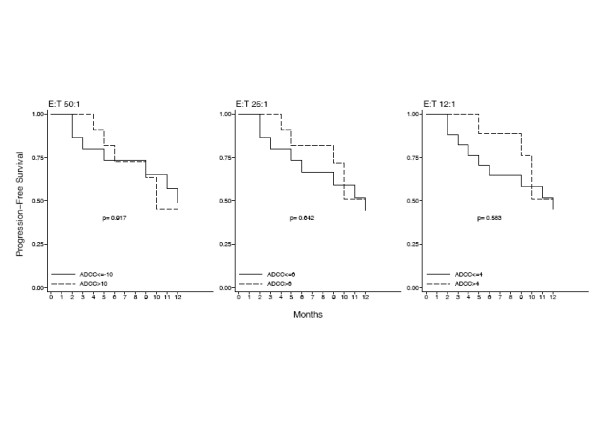
**Time to tumor progression is not correlated with the ADCC activity**. A cut-off of 10%, 6% and 4% cytotoxicity was arbitrarly chosen to discriminate between high and low levels ADCC at the three E:T ratios of 50:1, 25:1 and 12:1 respectively and a stratified PFS curve was created from these values. The pattern of the two curves and the Log-Rank test *p *values demonstrated that time to tumor progression was not correlated with the ADCC.

The Kaplan-Meier PFS curve showed that 25% and 50% of patients were in PD at 6 and 12 months respectively (Fig. 5). ROC analysis was used to provide an optimal cut-off for discrimination of high an low NK and ADCC activities at the three E:T ratios and stratified PFS curves were created. Results show that patients with higher levels of NK activity are significantly less likely to relapse within twelve months (Log-Rank test p-values 0.168, 0.033 an 0.024 at the 50:1, 25:1 and 12:1 E:T ratios respectively (Fig. 6). By contrast, the advantage of a high ADCC activity is only evident at six months. At twelve months, the percentage of relapsing patients with either a high or a low initial ADCC activity is 50% (Fig. 7).

## Discussion

Patients with Her2-overexpressing breast tumors normally become resistant to trastuzumab after one year. Hence, in addition to protein overexpression and gene amplification of Her2, other predictive response markers are needed to optimize its clinical use.

Since NK cell-mediated killing of the mAb-coated Her2-positive autologous tumor is part of the mechanism of action of trastuzumab, the functional state of NK cells may also critically influence the clinical outcome. We have therefore correlated this pretreatment biomarker with clinical response to trastuzumab after 6 months and 12 months. Results show that the short-term response rests on efficient NK and ADCC functions, whereas the long-term response is correlated with high NK function, but is independent of the ADCC profile.

In vitro studies [17,19] have demonstrated that NK cells kill trastuzumab-coated Her2-overexpressing cells via a FcγRIII receptor-mediated ADCC mechanism. This activity is very likely to recapitulate the destiny of the host tumor cells upon trastuzumab administration. Indeed, the significance of antibody-mediated killing in the clinical effect of humanized mAbs has been confirmed by clinical data [26]. In particular, experimental [17,19] and clinical [21,22] data point to the importance of the FcγR in trastuzumab's clinical efficiency PBMC from trastuzumab-treated patients displayed cytopathic activity in vitro against Her2-overexpressing cells. ADCC activity was more pronounced in tumours demonstrating a good response [21]. Moreover, increased infiltration of NK cells has been observed at the tumor site after trastuzumab [22]. However, evaluation of ADCC in metastatic breast cancer patients treated with a combination of trastuzumab and interleukin-2 (IL-2) [20,26] or IL-12 [27] (two activators of NK cells) did not confirm a correlation with clinical responses.

Our data show that patients with normal levels of both NK and ADCC activities respond more favourably to trastuzumab after a six month observation.

The ADCC performance status seems to depend on individual-related characteristics [28] such as FcγR polymorfisms [28,29], as well as the relative frequency of FcγR (CD16+)CD3+ vs CD56+CD3- NK subpopulations [[29] and our unpublished data], rather than on the individual trastuzumab pharmacokinetics [29].

Whatever the mechanisms that determine an effective ADCC response in trastuzumab- responsive patients, this was confined in our study to the early observation period.

Longer evaluation of the response to trastuzumab, in terms of progression-free survival after twelve months showed a strong correlation with the levels of NK activity only. By this time 75% of patients with low NK activity had relapsed, whereas the same percentage of those with high NK activity were still progression-free. By contrast, 50% of patients who had progressed after twelve months could not been distinguished through their ability to perform ADCC in vitro. This suggests that NK cells can protect against breast cancer progression in an antibody-independent fashion, a likely mechanism being targeting of immunoedited MHC class I defective tumor cells [30].

Taken as a whole, our data show that the efficiency of the NK system may be a hallmark of responsiveness to trastuzumab and suggest that breast cancer patients eligible for its administration may benefit from enhancement of this system in vivo. This includes combination therapy with trastuzumab and immunomodulatory agents or construction of bispecific antibodies targeting HER2 and CD16 [31] The first approach seems the more feasible. The fact that in vivo NK cell expansion and increased in vitro ADCC by IL-2 [20] or IL-12 [26,27] did not result in evident clinical benefits may merely mean that optimal combination of NK stimulators has still be found. A NK stimulation threshold, represented by a combination of IL-2 and IL-12 activates both the ADCC function and release of the antitumor cytokines and chemokines and is prone to be applied to clinical models [32]. IL-21 is also a promising NK stimulator, since it enhanced the effects of cetuximab in a murine tumor model and the human NK cell response to Ab-coated targets [33]. Once the mechanisms of ADCC function and the populations involved are characterized adoptive transfer of ex-vivo expanded purified ADCC effectors could be envisaged.

## Conclusion

Our finding that only patients who respond to trastuzumab after a six month observation have normal levels of NK and ADCC activities point to an important contribution of the host NK competence to the clinical response. The observation that longer (12 months) protection against tumor expansion is only associated with classical NK activity suggests that NK-associated mechanisms other than ADCC. are involved in long-term trastuzumab activity. A better prediction of trastuzumab response in individual patients may derive from the understanding of these mechanisms.

## List of abbreviations

NK: Natural Killer; ADCC: Antibody Dependent Cell Cytotoxicity; PFS: Progression-Free Survival.

## Competing interests

The authors declare that they have no competing interests.

## Authors' contributions

LM had the idea for, coordinated and analysed experimental data, and wrote the report.

AB contributed to protocol design, patients enrolment, follow up and clinical care

DM and LC contributed to protocol design

MM, MAP and contributed to follow up and clinical care.

ES performed the NK and ADCC tests

DB performed flowcytometric analysis

AE performed statistical analysis
